# Symptoms of and coping strategies for music performance anxiety through different time periods

**DOI:** 10.3389/fpsyg.2023.1138922

**Published:** 2023-05-31

**Authors:** Nanako Irie, Yuki Morijiri, Michiko Yoshie

**Affiliations:** ^1^Department of Information Technology and Human Factors, National Institute of Advanced Industrial Science and Technology (AIST), Tsukuba, Japan; ^2^Graduate School of Education, Yokohama National University, Yokohama, Japan; ^3^Graduate School of Education, Tokyo Gakugei University, Tokyo, Japan

**Keywords:** music performance anxiety, stage fright, symptom, coping strategy, musician, pianist, emotion

## Abstract

Music performance anxiety (MPA) manifests itself at mental, physiological, and behavioral levels. The present study investigated how the experience of the three levels of symptoms changes over time, and how musicians cope with these temporal changes in MPA symptoms. To this end, we conducted a questionnaire survey in which 38 student musicians freely commented on their experiences of mental and physical changes, as well as their coping strategies for these changes. This was examined during five different time periods around public performance, extending from the beginning of the preparation for a public performance until shortly before the next public performance. The free-text comments obtained from the questionnaire were analyzed thematically and classified into different response themes. We then examined the temporal changes in the frequency of comments on each response theme. We further conducted a semi-structured interview involving eight musicians to explore the responses to the questionnaire in greater detail. We analyzed the contents of the free-text comments obtained from the questionnaire and the interview for each response theme, focusing on the most frequently mentioned sub-themes. The results indicate that musicians started to experience mental MPA symptoms (e.g., negative feelings) as soon as they began to prepare for public performance. To cope with mental symptoms, musicians employed mental strategies such as positive thinking/self-talk and concentration both before and during public performance. The experience of physiological MPA symptoms (e.g., increased heart rate) peaked shortly before public performance and remained throughout performance. To cope with a variety of physiological symptoms, musicians employed physical strategies, especially deep breathing and exercise, shortly before public performance. In contrast, behavioral MPA symptoms (e.g., tremor) were experienced mostly during public performance. Some musicians also reported experiencing the actual impairment of performance quality. To avoid this, musicians employed a variety of practicing techniques (e.g., playing at a slower tempo) during the preparation for public performance and performing techniques (e.g., paying attention to expressions) during public performance. Together, the present findings indicate that mental, physiological, and behavioral symptoms of MPA exhibit differential timelines and that musicians effectively utilize different coping strategies according to the temporal changes in MPA symptoms.

## 1. Introduction

Music performance anxiety (MPA) is a significant problem for most music performers. MPA has been defined as “the experience of persisting, distressful apprehension about and/or actual impairment of, performance skills in a public context, to a degree unwarranted given the individual’s musical aptitude, training, and level of preparation” (Salmon, 1990, p. 3). Previous surveys indicate a high prevalence of MPA among musicians (Fishbein et al., 1988; Wesner et al., 1990; van Kemenade et al., 1995; Yoshie et al., 2011). For example, 58.7% of professional musicians playing in symphonic orchestras in the Netherlands reported experiencing MPA (van Kemenade et al., 1995). In another survey, 63.9% of Japanese classical musicians reported being troubled with MPA (Yoshie et al., 2011). MPA afflicts not only professional but also student musicians (Kaspersen and Gotestam, 2002; Fehm and Schmidt, 2006; Zakaria et al., 2013; Paliaukiene et al., 2018; Zarza-Alzugaray et al., 2018; Sickert et al., 2022). A recent survey found that 96% of undergraduates studying music were suffering from MPA (Zakaria et al., 2013). Moreover, 36.5% of music students reported a need for help with MPA (Kaspersen and Gotestam, 2002). MPA negatively influences musicians’ careers (van Kemenade et al., 1995; Fehm and Schmidt, 2006), and can sometimes force student musicians to abandon their musical studies (Orejudo Hernández et al., 2018). In light of the prevalence and severity of the problem, it is important for young student musicians to develop and establish their own highly effective coping strategies for MPA.

The symptoms of MPA are categorized into three groups: mental, physiological, and behavioral (Salmon, 1990; Steptoe, 2001; Burin and Osório, 2017). The *mental* symptoms involve the experience of negative emotions such as anxiety and cognitive problems such as memory slips (Steptoe, 2001; Yoshie et al., 2008b, 2009c). The *physiological* symptoms involve changes in physiological states, such as increased heart rate and perspiration (Salmon, 1990; Steptoe, 2001; Yoshie et al., 2008a, 2009a,b). The *behavioral* symptoms involve visible changes in musicians’ behavior, such as tremor and the impairment of performance quality (Steptoe, 2001; Yoshie et al., 2008a, 2009a,b; Burin and Osório, 2017; Kotani and Furuya, 2018).

Previous studies examining the three types of MPA symptoms mainly focused on the mental or physical changes that appear shortly before or during public performance (Brotons, 1994; Yoshie et al., 2008a,b, 2009a,c; Kotani and Furuya, 2018). However, several pieces of evidence suggest that MPA symptoms can persist for a longer period of time around public performance. The survey by van Kemenade et al. (1995) demonstrated that professional musicians can suffer from MPA weeks or even months prior to a performance. More recently, Zakaria et al. (2013) showed that most student musicians experience MPA before and during public performance, but that some of them also experience it after performance. Furthermore, Papageorgi et al. (2013) demonstrated that the level of MPA reaches a peak shortly before public performance, and decreases during performance. Although these studies effectively show that the level of MPA can vary across different time periods around public performance, it is still unknown how the mental, physiological, and behavioral symptoms of MPA change over time.

To address this, we adopted a qualitative approach in which musicians’ responses to open questions in a questionnaire survey and those in a semi-structured interview were analyzed thematically. Previous surveys on MPA typically asked musicians to answer a fixed set of closed questions to indicate how often (or how strongly) they experienced each MPA symptom, and their responses were analyzed quantitatively and often statistically (Wolfe, 1989; Kenny et al., 2004; Osborne and Kenny, 2005; Yoshie et al., 2008b, 2009c; Kobori et al., 2011). This quantitative approach has been successfully elucidating psychological mechanisms underlying MPA. However, the approach contains a limitation in that it does not allow researchers to determine which of the three types of MPA symptoms dominates within a musician’s subjective experience during a given time period around public performance. We thus asked student musicians to describe the mental and physical changes that they experienced during five different time periods around public performance. The contents of the free-text comments from the respondents were then analyzed to identify different response themes, including the three types of MPA symptoms. As in previous qualitative research (Franzosi, 2004; Alhija and Fresko, 2009; Maisonneuve et al., 2014), we analyzed the frequency of comments within each response theme to investigate the timelines of the three types of MPA symptoms around public performance. We then analyzed the contents of comments with a focus on the most frequently mentioned sub-themes for each response theme.

Previous literature suggests that musicians employ a variety of coping strategies to mitigate MPA, such as self-talk, cognitive restructuring, deep breathing, and practicing techniques (Fehm and Schmidt, 2006; Su et al., 2010; Studer et al., 2011; Hoffman and Hanrahan, 2012; Zhukov, 2019; Huang and Song, 2021). A recent study effectively demonstrated that musicians choose to use different coping strategies in different time periods around public performance (Huang and Song, 2021). However, it is still unclear whether/how musicians modulate their coping strategies according to the timelines of mental, physiological, and behavioral MPA symptoms. To address this, the questionnaire also asked student musicians to describe how they coped with the mental/physical changes during different time periods around public performance. We then analyzed the free-text comments thematically with an inductive (i.e., codebook) approach (Braun and Clarke, 2006). This approach enabled us to identify three response themes for coping strategies: mental, physical, and performance strategies. We analyzed the frequency of comments within each response theme to investigate the timelines of the three types of coping strategies around public performance. Here again, we analyzed the contents of comments with a focus on the most frequently mentioned sub-themes for each response theme.

Since semi-structured interviews are useful for exploring participants’ thoughts and feelings about a specific topic in detail (Dejonckheere and Vaughn, 2019), we further conducted a semi-structured interview involving both student and professional musicians. In the three-hour interview sessions, we asked participants to give us more details about their experiences related to MPA symptoms and coping strategies. We also compared the interview data between student and professional musicians to examine whether there were any differences in the experience of MPA symptoms and related coping strategies.

## 2. Materials and methods

### 2.1. Participants

Thirty-eight undergraduate and postgraduate students (34 females, *M*_age_ ± *SD* = 20.5 ± 1.7 years) majoring in music performance (12 students) or music education (26 students) at two universities in Japan participated in a questionnaire survey. Out of the 38 students, 29 reported majoring in piano, three reported majoring in singing and piano, three reported majoring in singing, two reported majoring in wind instruments, and one reported majoring in a string instrument. On average, participants started to play their major instrument at the age of 5.7 ± 4.2 years.

Since a previous survey in Japan demonstrates that pianists are most likely affected by MPA among different types of musicians (Yoshie et al., 2011), we focused on pianists in a following semi-structured interview. Participants were chosen from the group of students majoring in music performance (12 students), which included nine pianists. Two out of the nine pianists who played popular music in addition to classical music were excluded, and the remaining seven potential interviewees played classical music only. Based on their availability and willingness to participate in a follow-up study, five female student pianists (*M*_age_ ± *SD* = 19.4 ± 2.1 years) took part in the interview to report more details about their experiences related to MPA symptoms and coping strategies. To compare the responses from students with those from more experienced musicians, we additionally invited three professional pianists (one female, *M*_age_ ± *SD* = 62.0 ± 1.7 years) to the semi-structured interview. The professional pianists had been performing for an average of 57.0 ± 1.0 years and had studied music performance both in Japan and in Europe. Since the available sample was small, we did not apply any additional exclusion criteria such as the use of medication.

This study was approved by the National Institute of Advanced Industrial Science and Technology (AIST) Ethics Committee. All participants gave written informed consent.

### 2.2. Design and procedure

#### 2.2.1. Questionnaire survey

We asked 38 participants to recall their past public performances in general and to answer two open-ended questions about these performances: (1) what changes in mental and physical states they experienced and (2) how they coped with these mental and physical changes. Participants gave answers to these two questions for five different time periods around public performance: (1) from the beginning of the preparation for a public performance until the day before the performance (*P1*), (2) from the morning of the performance day until shortly before going onstage (*P2*), (3) during the public performance (*P3*), (4) from shortly after going offstage until the end of the performance day (*P4*), and (5) from the next day of the performance until shortly before the next public performance (*P5*).

#### 2.2.2. Semi-structured interview

We interviewed each of the eight participants (five students and three professional pianists) in either one three-hour session or in two 90-min sessions. In the semi-structured interview, we asked all participants the same questions in the same order to ensure that the data collected were comparable (McIntosh and Morse, 2015), although participants were allowed flexibility in responding to the questions. As in the questionnaire survey, we asked participants: (1) what changes in mental and physical states they experienced and (2) how they coped with these mental and physical changes. During the interview, participants could refer to a list of typical MPA symptoms derived from previous literature. The interview also included participants’ brief history of piano performance and the transitions of MPA in their performing life. All the interviews were conducted in Japanese and audio-recorded with the permission of participants.

### 2.3. Data analyses

#### 2.3.1. Qualitative analyses

For both the questionnaire and the interview, all comments regarding the changes in mental and physical states were transcribed and then analyzed thematically. The comments were classified into five response themes: (1) mental (i.e., cognitive or emotional) MPA symptoms, (2) physiological MPA symptoms, (3) behavioral MPA symptoms, (4) positive states/changes, and (5) others, based on previous literature (Salmon, 1990; Steptoe, 2001; Yoshie et al., 2011; Burin and Osório, 2017). Subsequent analyses focused on the first four response themes. Although some of the reported symptoms (e.g., tremor) could be viewed as both physiological and behavioral (Wesner et al., 1990; Steptoe, 2001; Burin and Osório, 2017; Fernholz et al., 2019), we classified all the visible changes in musicians’ behavior as behavioral MPA symptoms (Steptoe, 2001; Burin and Osório, 2017).

All comments about coping strategies toward MPA were analyzed thematically as an inductive approach. In the process of the thematic analysis, we applied a codebook approach using an initial codebook with themes derived from previous literature and developed it to generate codes and themes from the data (Braun and Clarke, 2006). The coded comments were progressively refined for further sub-themes based on the coping strategies defined in previous literature. As a result, three response themes were identified for coping strategies: (1) mental strategies, (2) physical strategies, and (3) performance strategies. The categorization criteria were as follows: First, any strategies that were directly related to music performance or practice were classified into *performance* strategies. Second, out of the remaining strategies, those aimed at modifying musicians’ physical state were classified into *physical* strategies. Third, since all the remaining strategies aimed at modifying musicians’ cognitive and/or emotional state, they were classified into *mental* strategies.

To ensure the validity of the classifications, one researcher classified the data into themes, and then the other two researchers overviewed and considered the outcomes. In the process of coding, all three researchers reviewed the generated themes and then ensured consistency in the decisions and interpretations made. When we needed to cite an example of a comment from the questionnaire or interview data, one researcher translated it into English. Subsequently, another researcher reviewed the translation, and the two researchers modified it together, when necessary.

#### 2.3.2. Quantitative analyses

For the questionnaire data, we counted the number of comments classified into each response theme for each of the five time periods (i.e., P1-P5) for each participant. The number of comments was first analyzed with two-way analyses of variance (ANOVA) of theme × period. Since the two-way interaction was found to be significant both for the mental/physical changes and coping strategies, we subsequently examined the interactions by performing a follow-up one-way ANOVA of the period for each theme. In all ANOVAs, Greenhouse–Geisser correction was applied to the degrees of freedom where the sphericity assumption was violated.

## 3. Results and discussion

This study aimed to investigate how the three types of MPA symptoms change over time around public performance and how musicians cope with these changes. To this end, we combined a questionnaire survey with a semi-structured interview to ask musicians to report their experiences of any mental/physical changes during five different time periods around past public performances and their strategies for coping with these changes.

### 3.1. Temporal changes in mental/physical states around public performance

In the questionnaire survey, the number of participants (out of the 38 student musicians) who wrote at least one comment on the mental, physiological, behavioral symptoms of MPA, or positive states/changes was 33, 21, 22, and 28, respectively (Table 1). Thus, student musicians recognized the mental symptoms most frequently out of the three types of MPA symptoms.

**Table 1:** Number of participants commenting on each theme of mental/physical changes in the questionnaire survey.

Theme	Sub-theme	Number (and percentage) of participants					
		P1	P2	P3	P4	P5	P1-P5
Mental symptoms	Negative feelings	20 (52.6)	23 (60.5)	10 (26.3)	7 (18.4)	3 (7.9)	**32 (84.2)**
	Cognitive problems	0 (0.0)	0 (0.0)	5 (13.2)	0 (0.0)	1 (2.6)	**6 (15.8)**
	**Total**	**20** **(52.6)**	**23** **(60.5)**	**14** **(36.8)**	**7** **(18.4)**	**4** **(10.5)**	**33** **(86.8)**
Physiological symptoms	Increased heart rate	0 (0.0)	13 (34.2)	6 (15.8)	1 (2.6)	0 (0.0)	**16 (42.1)**
	Perspiration	0 (0.0)	5 (13.2)	2 (5.3)	0 (0.0)	0 (0.0)	**7 (18.4)**
	Cold hands	0 (0.0)	3 (7.9)	1 (2.6)	0 (0.0)	0 (0.0)	**4 (10.5)**
	Gastrointestinal disturbances	1 (2.6)	3 (7.9)	0 (0.0)	0 (0.0)	0 (0.0)	**4 (10.5)**
	Sleep disturbances	1 (2.6)	0 (0.0)	0 (0.0)	0 (0.0)	0 (0.0)	**1 (2.6)**
	Hot flash	0 (0.0)	0 (0.0)	1 (2.6)	0 (0.0)	0 (0.0)	**1 (2.6)**
	**Total**	**2** **(5.3)**	**17** **(44.7)**	**9** **(23.7)**	**1** **(2.6)**	**0** **(0.0)**	**21** **(55.3)**
Behavioral symptoms	Tremor	0 (0.0)	3 (7.9)	10 (26.3)	3 (7.9)	0 (0.0)	**13 (34.2)**
	Muscle stiffness	1 (2.6)	1 (2.6)	5 (13.2)	0 (0.0)	0 (0.0)	**6 (15.8)**
	Impairment of performance quality	1 (2.6)	0 (0.0)	3 (7.9)	0 (0.0)	0 (0.0)	**4 (10.5)**
	Overtraining	2 (5.3)	0 (0.0)	0 (0.0)	0 (0.0)	1 (2.6)	**3 (7.9)**
	Floating feeling	0 (0.0)	0 (0.0)	1 (2.6)	1 (2.6)	0 (0.0)	**1 (2.6)**
	**Total**	**4** **(10.5)**	**3** **(7.9)**	**17** **(44.7)**	**4** **(10.5)**	**1** **(2.6)**	**22** **(57.9)**
Positive states/changes	Positive feelings	3 (7.9)	2 (5.3)	4 (10.5)	21 (55.3)	9 (23.7)	**24 (63.2)**
	Disappearance of MPA symptoms	0 (0.0)	0 (0.0)	4 (10.5)	3 (7.9)	0 (0.0)	**7 (18.4)**
	Improvement of concentration	0 (0.0)	0 (0.0)	3 (7.9)	0 (0.0)	0 (0.0)	**3 (7.9)**
	Positive behaviors	1 (2.6)	0 (0.0)	2 (5.3)	0 (0.0)	0 (0.0)	**3 (7.9)**
	**Total**	**4** **(10.5)**	**2** **(5.3)**	**11** **(28.9)**	**23** **(60.5)**	**9** **(23.7)**	**28** **(73.7)**

The values in parentheses show the percentages of participants within the present sample. The bold “Total” values indicate the numbers (and percentages) of participants commenting on at least one of the sub-themes for each theme. The bold “P1-P5” values indicate the numbers (and percentages) of participants commenting for at least one of the time periods. P1, time period 1 (from the beginning of the preparation for a public performance until the day before the performance); P2, time period 2 (from the morning of the performance day until shortly before going onstage); P3, time period 3 (during the public performance); P4, time period 4 (from shortly after going offstage until the end of the performance day); P5, time period 5 (from the next day of the performance until shortly before the next public performance).

**Figure 1:**
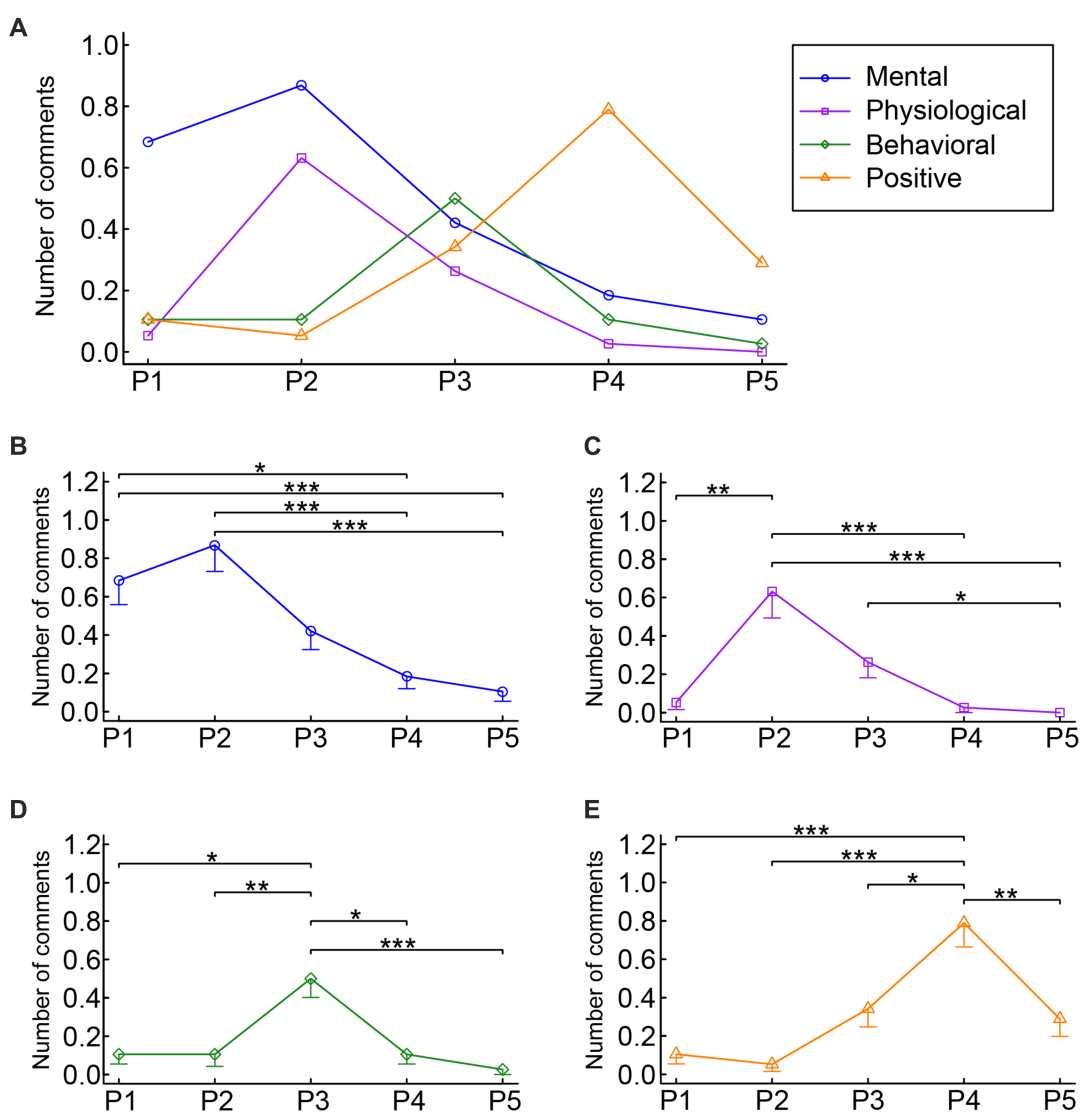
Mean number of comments for mental, physiological, behavioral MPA symptoms and positive states/changes. **(A)** Summary of the results. **(B–E)** Results of the statistical analyses for mental, physiological, behavioral MPA symptoms and positive states/changes, respectively. * *p* < 0.05, ** *p* < 0.01, *** *p* < 0.001. The error bars represent standard errors of the mean. P1: time period 1 (from the beginning of the preparation for public performance until the day before the performance). P2: time period 2 (from the morning of the performance day until shortly before going onstage). P3: time period 3 (during the public performance). P4: time period 4 (from shortly after going offstage until the end of the performance day). P5: time period 5 (from the next day of the performance until shortly before the next public performance).

A two-way ANOVA of theme × period on the number of comments (Figure 1A) revealed a significant interaction effect (*F* (6.58, 243.36) = 11.41, *p* < 0.001, partial η^2^ = 0.24), in addition to a significant main effect of the theme (*F* (2.54, 93.86) = 9.52, *p* < 0.001, partial η^2^ = 0.21) and of the period (*F* (3.61, 133.59) = 17.12, *p* < 0.001, partial η^2^ = 0.32). The follow-up one-way ANOVAs for each of the four themes revealed a significant main effect of the period for mental MPA symptoms (*F* (2.81, 103.86) = 11.62, *p* < 0.001, partial η^2^ = 0.24), physiological MPA symptoms (*F* (1.80, 66.76) = 13.73, *p* < 0.001, partial η^2^ = 0.27), behavioral MPA symptoms (*F* (2.57, 95.12) = 9.70, *p* < 0.001, partial η^2^ = 0.21), and positive states/changes (*F* (2.89, 106.71) = 13.44, *p* < 0.001, partial η^2^ = 0.27), respectively. Therefore, we subsequently conducted *post hoc* Bonferroni multiple comparisons for each of the four themes to explore the origin of the main effect.

#### 3.1.1. Mental MPA symptoms

The questionnaire data showed that the number of comments on mental MPA symptoms peaked in P2, gradually decreasing toward P5 (Figure 1B). The *post hoc* multiple comparisons for mental MPA symptoms demonstrated that the number of comments for P1 was significantly greater than that for P4 (*p* = 0.019) and P5 (*p* < 0.001). The number of comments for P2 was also greater than that for P4 (*p* < 0.001) and P5 (*p* < 0.001). These results suggest that mental MPA symptoms were recognized by student musicians mainly *before* public performance. All the comments for P1 (20 participants) and P2 (23 participants) mentioned the experience of negative feelings such as anxiety and nervousness (Table 1). For example:

“As the performance day approaches, I start to imagine the performance situation and get nervous little by little.” (a student)

In P3, 10 participants reported experiencing negative feelings and five participants reported experiencing cognitive problems such as memory slips (Table 1). A typical example of memory slips was as follows:

“I sometimes forget what I should play next during a public performance.” (a student)

A few participants experienced negative feelings such as anxiety and depression even after public performance (P4 and P5). This timeline complies with previous findings indicating that most musicians experience MPA before and during public performance (Papageorgi et al., 2013; Zakaria et al., 2013).

Consistent with the results of the questionnaire, the interview participants also reported experiencing negative feelings such as anxiety and nervousness mainly in P1 and P2. The interview data indicated that there were two psychological factors underlying their negative feelings: the concern over mistakes and the fear of negative evaluation by others. All five students reported that they worried about making mistakes, which exacerbated their anxiety. For example:

“I was worried about mistakes. Well, I made mistakes on the certain part where I did not want to make mistakes. If I make mistakes at the climax part or conspicuous part, I feel ‘Oh no’…” (a student)

“I could not imagine my performance would be going well. I seriously thought that I should not make mistakes.” (a student)

All five students were also sensitive about how their performance would be evaluated by their teachers, family, friends, colleagues, or audiences. For example:

“(describing a competition) As even I slightly feel like, ‘Oh, that person’s performance has changed from before,’ I understand that I could be thought of like this by others, too. So, I do not like for people to think, ‘Her performance sounds like she did not practice enough this time’ or something like this… well, [I worry about] evaluation from others.” (a student)

“I unintentionally pressured myself when I started thinking how much my parents did to lead me to this stage; for example, driving for a couple of hours for my piano competition… I did not think until the public performance, but I realized on the performance day that I got more nervous than I thought.” (a student)

Previous questionnaire surveys indicate that perfectionistic thinking, especially the concern over mistakes, increases subjective anxiety or distress in musicians (Stoeber and Eismann, 2007; Yoshie and Shigemasu, 2007; Kobori et al., 2011). Furthermore, the interaction between the concern over mistakes and the fear of negative evaluation by audiences was found to further exacerbate subjective anxiety in pianists (Yoshie and Shigemasu, 2007). The interview data obtained from student musicians are consistent with these previous findings. Meanwhile, none of the professional pianists mentioned making mistakes or negative evaluations as being a serious issue, which may have been helpful in alleviating their anxiety.

Four students and two professionals reported having anxiety over memorizing music in P1 and P2. For example:

“Playing from memory is not the first thing to worry about, but it is always an underlying worry for a performance.” (a professional pianist)

Two students and all three professionals also reported having experienced memory slips as a cognitive symptom in P3. For example:

“It was traumatic. I stopped playing (during the performance) and my mind went blank… It became traumatic that I actually forgot the piece and stopped playing.” (a student)

These comments suggest that memorizing music is another source of anxiety in public performance.

In P4 and P5, although student musicians reported experiencing burnout, professionals could switch their feelings shortly after public performance. Compared to professionals, student musicians tended to experience mental MPA symptoms for a longer period of time, extending from the beginning of the preparation for a public performance until days or even weeks after the performance.

#### 3.1.2. Physiological MPA symptoms

The questionnaire data showed that the number of comments on physiological MPA symptoms peaked in P2, gradually decreasing toward P5 (Figure 1C). The *post hoc* multiple comparisons for physiological MPA symptoms demonstrated that the number of comments for P2 was significantly greater than that for P1 (*p* = 0.003), P4 (*p* < 0.001), and P5 (*p* < 0.001), respectively. In addition, the number of comments for P3 was significantly greater than that for P5 (*p* = 0.026). Therefore, physiological MPA symptoms were mainly recognized *shortly before* and *during* public performance. The most frequently mentioned symptom was increased heart rate, with 13 participants reporting it in P2 and 6 participants in P3 (Table 1). For example:

“My heart beats faster just before public performance.” (a student)

Other frequently mentioned symptoms included perspiration, cold hands, and gastrointestinal disturbances (Table 1).

In the interview, all five students and two professionals reported experiencing increased heart rate mainly in P2 and P3. For example:

“I often feel that my heart beats faster… Definitely heart beats get faster, absolutely. Absolutely it happens, of course.” (a professional pianist)

The results show that most participants recognized increased heart rate shortly before and during public performance. This finding is consistent with previous psychophysiological experiments demonstrating that musicians’ heart rate dramatically increases during public performance compared to practice or rehearsals (Fredrikson and Gunnarsson, 1992; Brotons, 1994; Yoshie et al., 2009a).

All five students and two professionals also reported experiencing cold hands/body. For example:

“My hands were cold.” (a student)

Four students and one professional pianist reported experiencing sleep and gastrointestinal disturbances. Notably, student musicians reported experiencing anorexia before public performance. For example:

“I could not sleep well when I felt that I did not practice enough.” (a professional pianist)

“I can eat in the morning, but just before a performance, I cannot eat much. I can manage to drink like jelly beverage, but for example, bread… maybe not.” (a student)

The physiological symptoms reported here were all consistent with those reported in previous studies (Salmon, 1990; Steptoe, 2001; Yoshie et al., 2008a, 2009a,b). Interestingly, both the experience of negative feelings and the recognition of physiological symptoms peaked shortly before public performance (Figures 1B,C), supporting previous findings (Papageorgi et al., 2013; Zakaria et al., 2013). This may highlight the important role of bodily responses in the experience of anxiety (Critchley, 2005). Two professionals looked back on their youth and reported that they used to have more physiological symptoms, such as sweaty palms and gastrointestinal disturbances. This complies with previous studies suggesting that many years of performance experience can sometimes lead to a reduction in MPA (Steptoe and Fidler, 1987; Osborne and Franklin, 2002).

Interestingly, student and professional musicians differed in the way they perceived physiological MPA symptoms. Student musicians tended to perceive physiological symptoms negatively, which could aggravate their subjective anxiety. For example:

“My hands get very cold before going onstage, but they get sweaty onstage. I then feel like ‘I want to go home’ almost every time…” (a student)

In contrast, professional musicians perceived physiological symptoms as an unharmful response, or even as a positive response that can enhance performance quality. For example:

“… well, I feel that my heart beats faster. But I would not make mistakes because of that. I just keep on going, taking it as it comes.” (a professional pianist)

“As for perspiration, I think it’s good to warm up my body until I start to sweat. Otherwise, my body would not move properly. So, it’s natural to sweat a bit.” (a professional pianist)

Previous literature suggests that physiological MPA symptoms can facilitate music performance in certain conditions (Hamann and Sobaje, 1983; Roland, 1994; Gannon, 2019). In particular, musicians with considerable experience and expertise are likely to properly control their physiological symptoms and utilize them to enhance focus, excitement, and performance quality (Roland, 1994; Gannon, 2019). This might explain why professional pianists reported viewing physiological MPA symptoms as a positive part of performing in the present interview.

Another difference between students and professionals was found in the onset of physiological symptoms. Although student musicians started to experience physiological symptoms during the preparation for public performance, professional pianists rarely recognized physiological symptoms in this time period. Therefore, similarly to mental symptoms, students tended to experience physiological symptoms for a longer time compared to professionals.

#### 3.1.3. Behavioral MPA symptoms

The questionnaire data showed that the experience of behavioral MPA symptoms peaked in P3. The *post hoc* multiple comparisons for behavioral MPA symptoms demonstrated that the number of comments for P3 was significantly greater than that for P1 (*p* = 0.01), P2 (*p* = 0.002), P4 (*p* = 0.01), and P5 (*p* < 0.001), respectively. The results indicate that behavioral MPA symptoms were mainly recognized *during* public performance. The most frequently mentioned symptom in P3 was tremor, with 10 participants mentioning it (Table 1). For example:

“I get so nervous that my hands and feet tremble.” (a student)

Some participants reported that they experienced tremor even shortly before (three participants) and shortly after (three participants) public performance (Table 1).

The second most frequently mentioned symptom in P3 was muscle stiffness, with five participants mentioning it (Table 1). For example:

“I feel that my body is stiff.” (a student)

Additionally, three participants reported experiencing the actual impairment of performance quality in P3 (Table 1). For example:

“Although I try to produce the same sounds as those during practice, my fingers would not move well.” (a student)

Supporting the results of the questionnaire, the most frequently mentioned behavioral symptoms in the interview were muscle stiffness, tremor, and the impairment of performance quality. All eight participants of the interview reported having experienced muscle stiffness. For example:

“My body became stiff. I also realized that it affected my tone quality, which sounded hard… I was told to relax my body, but my shoulder was so stiff. I had no idea how to relax my body and release body tension.” (a student)

Furthermore, all five students and one professional pianist reported having experienced tremor. For example:

“My foot was trembling on the pedal.” (a professional pianist)

Both students and professional pianists reported experiencing these behavioral MPA symptoms in P3, but only students reported experiencing them in P2 as well.

Furthermore, all eight participants of the interview reported that their performance quality was sometimes impaired in P3. They reported making a variety of technical and artistic mistakes, such as melodic inaccuracies, unbalanced rhythm, rapid tempo, and inappropriate tone quality. For example:

“I played a wrong note at the end… that was the biggest mistake… my body was stiff, and the tone color was hard, too…” (a student)

“My performance got faster and faster… as like slipping. I felt like sliding on the ice.” (a professional pianist)

The behavioral symptoms reported here were consistent with those reported in previous studies (Salmon, 1990; Steptoe, 2001; Yoshie et al., 2008a, 2009a,b; Kotani and Furuya, 2018). Both tremor and muscle stiffness can have detrimental effects on performance quality. Involuntary tremor of various body parts (e.g., hands, fingers, and feet) would disturb the fine motor control of musicians. Muscle stiffness, which could be related to heightened muscle activity and increased co-contraction levels of antagonistic muscles (Yoshie et al., 2009a), can adversely affect musical expressions by disrupting the subtle control of loudness (Yoshie et al., 2008a) and the maintenance of temporal continuity (Drake and Palmer, 2000). These behavioral symptoms would thus lead to a decrease in the sense of control (Guyon et al., 2022) and the actual impairment of performance quality (Yoshie et al., 2009a; Sokoli et al., 2022) during public performance.

Interestingly, behavioral MPA symptoms followed a timeline that was independent from the timeline of mental or physiological symptoms. The behavioral symptoms were recognized most frequently onstage, whereas mental and physiological symptoms were recognized most frequently shortly before public performance.

Here again, we found some differences in the experience of behavioral MPA symptoms between students and professionals. First, similarly to mental and physiological symptoms, students experienced behavioral symptoms for a longer time than did professionals: although professionals experienced behavioral symptoms only onstage, students tended to experience them throughout the performance day. Second, similarly to physiological symptoms, students perceived behavioral symptoms more negatively compared to professionals. Notably, students tended to perceive the impairment of performance quality as traumatic, while professionals felt that some mistakes are common and acceptable during public performance. The way of perceiving behavioral symptoms specific to students could aggravate their subjective anxiety.

#### 3.1.4. Positive states/changes

The number of comments on positive states/changes peaked in P4 (Figure 1E). The *post hoc* multiple comparisons for positive states/changes showed that the number of comments for P4 was significantly greater than that for P1 (*p* < 0.001), P2 (*p* < 0.001), P3 (*p* = 0.037), and P5 (*p* = 0.003), respectively. Therefore, the experience of MPA symptoms was replaced by that of positive states/changes shortly after public performance. In P4, 21 out of the 38 participants reported experiencing positive feelings such as relief and achievement (Table 1). For example:

“I feel relieved when a performance ends without any accidents.” (a student)

The experience of similar positive feelings was also reported by nine participants in P5. Furthermore, three participants reported the disappearance of MPA symptoms in P4 (Table 1). For example:

“I feel myself letting go of tension.” (a student)

Notably, 11 participants reported experiencing positive states/changes even in P3 or during public performance (Table 1). Their comments included the experience of positive feelings (four participants), the disappearance of MPA symptoms (four participants), and the improvement of concentration (three participants). For example:

“I enjoy music performance.” (a student)

“I get into my own world.” (a student)

The interview data indicate that both students and professionals could perceive a public performance as a positive experience if they could concentrate on the music itself in P3. For example:

“That performance relatively went well. I did not make any significant mistakes. Well, I mean, I did not feel that I messed up the performance with obvious mistakes.” (a professional pianist)

“Well, I felt a sense of unity in the hall when my performance went well. If I feel like this, it means I am satisfied with the performance.” (a professional pianist)

These results indicate that the experience of MPA symptoms is normally replaced by positive feelings when musicians go offstage. In some cases, this occurs as soon as musicians start to perform onstage, leading them to be in the “flow” state and to achieve optimal performance (Kirchner et al., 2008; Fullagar et al., 2013).

### 3.2. Temporal changes in coping strategies for MPA

In the questionnaire survey, the number of participants who wrote at least one comment on the mental, physical, and performance strategies for MPA was 22, 24, and 35, respectively (Table 2). Thus, student musicians employed performance strategies most frequently out of the three types of coping strategies.

**Table 2:** Number of participants commenting on each theme of coping strategies in the questionnaire survey.

Theme	Sub-theme	Number (and percentage) of participants					
		P1	P2	P3	P4	P5	P1-P5
Mental strategies	Positive thinking/self-talk	6 (15.8)	9 (23.7)	4 (10.5)	2 (5.3)	1 (2.6)	**15 (39.5)**
	Concentration	1 (2.6)	3 (7.9)	6 (15.8)	3 (7.9)	0 (0.0)	**12 (31.6)**
	Avoidance behaviors	1 (2.6)	1 (2.6)	1 (2.6)	1 (2.6)	0 (0.0)	**4 (10.5)**
	Ritualized behaviors	0 (0.0)	0 (0.0)	2 (5.3)	0 (0.0)	0 (0.0)	**2 (5.3)**
	**Total**	**8** **(21.1)**	**12** **(31.6)**	**10** **(26.3)**	**6** **(15.8)**	**1** **(2.6)**	**22** **(57.9)**
Physical strategies	Breathing	0 (0.0)	8 (21.1)	5 (13.2)	1 (2.6)	0 (0.0)	**14 (36.8)**
	Sleep/rest	4 (10.5)	2 (5.3)	0 (0.0)	1 (2.6)	4 (10.5)	**7 (18.4)**
	Exercise	0 (0.0)	6 (15.8)	0 (0.0)	0 (0.0)	0 (0.0)	**6 (15.8)**
	Wiping sweat	0 (0.0)	4 (10.5)	0 (0.0)	0 (0.0)	0 (0.0)	**4 (10.5)**
	Keeping hands/body warm	0 (0.0)	4 (10.5)	0 (0.0)	0 (0.0)	0 (0.0)	**4 (10.5)**
	Diet	2 (5.3)	1 (2.6)	0 (0.0)	0 (0.0)	1 (2.6)	**4 (10.5)**
	General physical conditioning	0 (0.0)	1 (2.6)	0 (0.0)	0 (0.0)	1 (2.6)	**2 (5.3)**
	Massage	1 (2.6)	0 (0.0)	0 (0.0)	0 (0.0)	0 (0.0)	**1 (2.6)**
	Body posture	0 (0.0)	0 (0.0)	1 (2.6)	0 (0.0)	0 (0.0)	**1 (2.6)**
	**Total**	**4** **(10.5)**	**19** **(50.0)**	**6** **(15.8)**	**2** **(5.3)**	**5** **(13.2)**	**24** **(63.2)**
Performance strategies	Content of practice	15 (39.5)	11 (28.9)	0 (0.0)	5 (13.2)	13 (34.2)	**24 (63.2)**
	Amount of practice	11 (28.9)	3 (7.9)	0 (0.0)	0 (0.0)	2 (5.3)	**15 (39.5)**
	Performing techniques	0 (0.0)	0 (0.0)	10 (26.3)	0 (0.0)	0 (0.0)	**10 (26.3)**
	Mental rehearsal	2 (5.3)	2 (5.3)	0 (0.0)	0 (0.0)	0 (0.0)	**4 (10.5)**
	**Total**	**26** **(68.4)**	**15** **(39.5)**	**10** **(26.3)**	**5** **(13.2)**	**15** **(39.5)**	**35** **(92.1)**

The values in parentheses show the percentages of participants within the present sample. The bold “Total” values indicate the numbers (and percentages) of participants commenting on at least one of the sub-themes for each theme. The bold “P1-P5” values indicate the numbers (and percentages) of participants commenting for at least one of the time periods. P1, time period 1 (from the beginning of the preparation for a public performance until the day before the performance); P2, time period 2 (from the morning of the performance day until shortly before going onstage); P3, time period 3 (during the public performance); P4, time period 4 (from shortly after going offstage until the end of the performance day); P5, time period 5 (from the next day of the performance until shortly before the next public performance).

**Figure 2:**
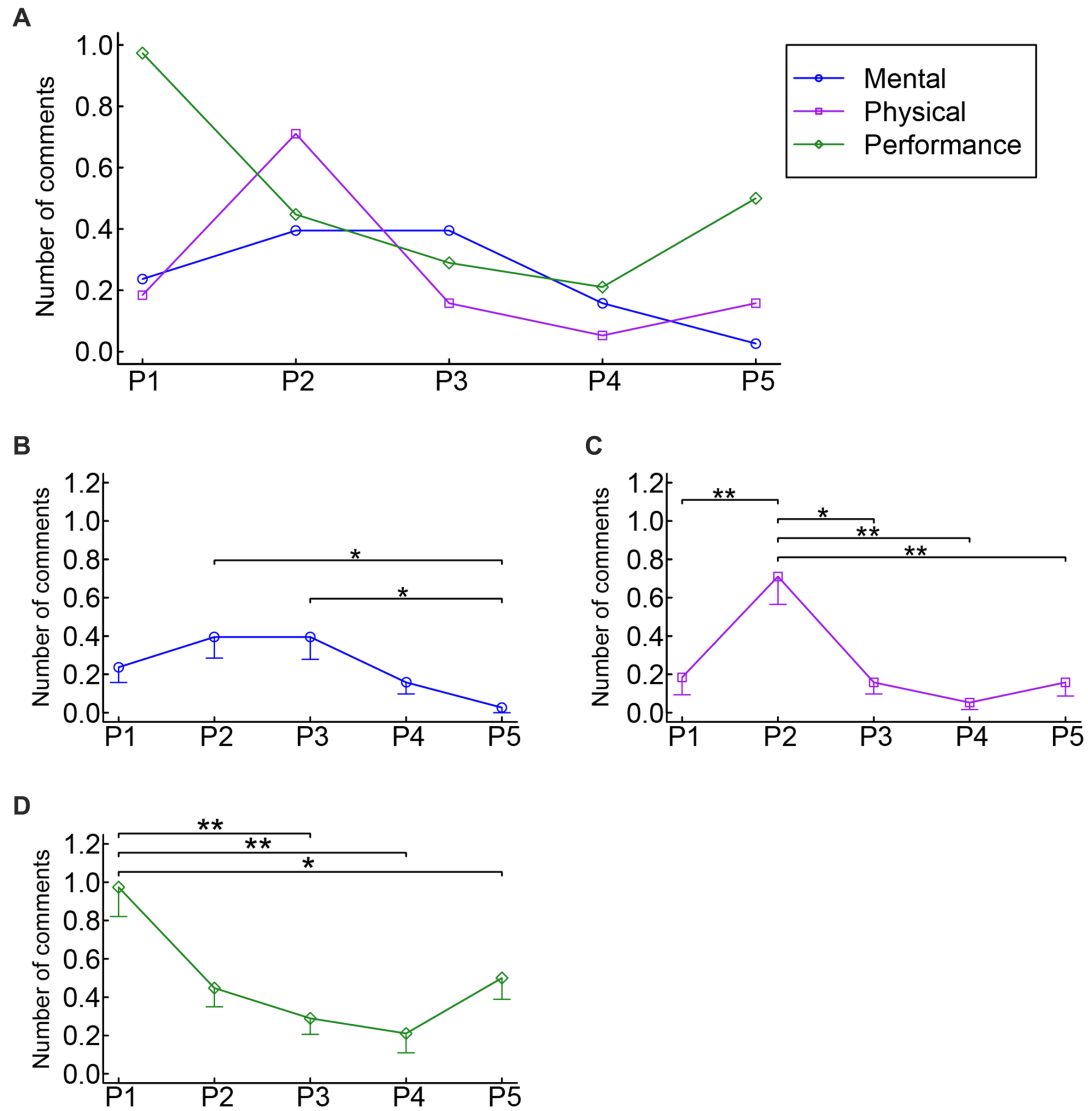
Mean number of comments for mental, physical, and performance strategies. **(A)** Summary of the results. **(B–D)** Results of the statistical analyses for mental, physical, and performance strategies, respectively. * *p* < 0.05, ** *p* < 0.01, *** *p* < 0.001. The error bars represent standard errors of the mean. P1: time period 1 (from the beginning of the preparation for public performance until the day before the performance). P2: time period 2 (from the morning of the performance day until shortly before going onstage). P3: time period 3 (during the public performance). P4: time period 4 (from shortly after going offstage until the end of the performance day). P5: time period 5 (from the next day of the performance until shortly before the next public performance).

A two-way ANOVA of theme × period on the number of comments (Figure 2A) revealed a significant interaction effect (*F* (5.44, 201.36) = 6.40, *p* < 0.001, partial η^2^ = 0.15), in addition to a significant main effect of the theme (*F* (1.75, 64.69 = 8.07, *p* = 0.001, partial η^2^ = 0.18) and of the period (*F* (2.84, 105.18) = 9.77, *p* < 0.001, partial η^2^ = 0.21). The follow-up one-way ANOVAs for each of the three themes revealed a significant main effect of the period for mental strategies (*F* (2.80, 103.55 = 3.85, *p* = 0.013, partial η^2^ = 0.09), physical strategies (*F* (2.38, 88.08) = 9.96, *p* < 0.001, partial η^2^ = 0.21), and performance strategies (*F* (2.68, 99.0) = 8.11, *p* < 0.001, partial η^2^ = 0.18), respectively. Therefore, we subsequently conducted *post hoc* Bonferroni multiple comparisons for each of the three themes to explore the origin of the main effect.

#### 3.2.1. Mental strategies for MPA

The questionnaire data showed that the number of comments on mental strategies was relatively stable throughout the five time periods investigated, but the number of comments for P2 and P3 was slightly greater compared to that in the other periods (Figure 2B). The *post hoc* multiple comparisons for mental strategies demonstrated that the number of comments for P5 was significantly smaller than that for P2 (*p* = 0.018) and P3 (*p* = 0.045), respectively. The results indicate that mental strategies for MPA were mainly employed *shortly before* and *during* public performance. The two most popular mental strategies in P2 were positive thinking/self-talk and concentration, with nine and three participants reporting them, respectively (Table 2). Typical examples of positive thinking/self-talk in P2 were as follows:

“I talk to myself, ‘I will be alright’.” (a student)

“I believe that the piece is not too difficult and that I am well-prepared for the performance.” (a student)

Six participants used the positive thinking/self-talk strategy also during the preparation for public performance (P1). For example:

“I talk to myself, ‘Since I have practiced intensely, I will be alright’.” (a student)

As in these examples, the positive thinking/self-talk strategy aimed to boost self-confidence.

The two most popular strategies in P3 were again concentration and positive thinking/self-talk, with six and four participants mentioning them, respectively (Table 2). A typical example of concentration was as follows:

“I concentrate on myself during my performance.” (a student)

The results are consistent with a recent survey demonstrating that positive self-talk, as well as instructional self-talk to boost concentration on performance, were intensely used in the early stage of preparation, backstage, and onstage by student musicians (Huang and Song, 2021). In addition, two of the present participants were engaged in ritualized behaviors such as putting on a charm in P3.

Corroborating the results of the questionnaire, all eight participants of the interview reported employing the positive thinking/self-talk strategy. This strategy helped the musicians to stay positive and to believe in themselves. For example, participants tried to convince themselves by pointing to the amount of preparation they had done for a public performance. For example:

“I talked to myself, ‘I have practiced many times’ to convince myself.” (a student)

“When I tried to relieve nervousness, I persuaded myself, like talking to myself, ‘I have practiced many times.’ I think I was using autosuggestion.” (a student)

The positive thinking/self-talk strategy also included cognitive restructuring. The following dialog in the interview illustrates how professional musicians perceived making mistakes during public performance in a positive way:

“[about making mistakes]…well, I’m a human being and [mistakes] can happen as like accidents. Audiences do not particularly expect a flawless performance.” (a professional pianist)

Some students also mentioned that conversation with their parents or teachers helped them think in a more positive way. For example:

“My mother makes me feel relieved a bit by saying ‘it’s not helpful to get nervous’.” (a student)

“My teacher advised me not to worry much and to play like myself, which was memorable.” (a student)

Previous empirical evidence suggests that positive thinking/self-talk can significantly reduce subjective anxiety and improve performance quality in musicians (Sweeney and Horan, 1982; Hoffman and Hanrahan, 2012). The present participants used the positive thinking/self-talk strategy most intensely shortly before public performance (P2), presumably to relieve negative feelings (e.g., anxiety) that were experienced most frequently in this time period (Figure 1B).

Interestingly, although student musicians employed both positive thinking and positive self-talk, professional pianists reported employing positive thinking only. Students seemed to be trying to divert their attention from undesirable thoughts and feelings effortfully by talking to themselves. In contrast, professionals tended to have positive and realistic thoughts more naturally, based on their considerable experience. For example:

“In my view, it is self-confidence [that matters]. After I have spent plenty of time in practicing carefully, I would just rely on what I have done. I will not be more than what I have done, but I am confident that I will not be less than that either.” (a professional pianist)

Furthermore, both students and professional pianists reported employing the concentration strategy as in the questionnaire. The example below indicates that concentration on performance itself can help musicians alleviate their anxiety:

“I think that I would not get nervous if I can get into my own world.” (a student)

Previous literature suggests that focusing on music performance by distracting oneself from surrounding task-irrelevant cues and/or the possibility of undesirable outcomes can reduce subjective anxiety and improve performance quality (Connolly and Williamon, 2004; Sutani and Akutsu, 2019). Therefore, the present participants employed the concentration strategy most intensely during public performance (P3), presumably to avoid the actual impairment of performance quality that was experienced in this time period (Figure 1D).

Consistent with the results of the questionnaire, some students reported being involved in ritualized behaviors in P3 to calm themselves down. For example:

“There is a handkerchief which I always take onstage… Before a performance, I rightly wipe the keyboard with it…” (a student)

Pre-performance routines, including ritualized behaviors as in this example, can help musicians be mentally well-prepared for public performance (Ginsborg, 2004; Klickstein, 2009).

#### 3.2.2. Physical strategies for MPA

The questionnaire data showed that the number of comments on physical strategies peaked in P2 (Figure 2C). The *post hoc* multiple comparisons for physical strategies demonstrated that the number of comments for P2 was significantly greater than that for P1 (*p* = 0.008), P3 (*p* = 0.010), P4 (*p* = 0.001), and P5 (*p* = 0.005), respectively. The results indicate that physical strategies for MPA were mainly employed *shortly before* public performance. The most popular physical strategy in P2 was breathing (e.g., deep breathing), with eight participants mentioning it (Table 2). For example:

“I try to take a deep breath.” (a student)

The breathing strategy was also employed in P3 by five participants (Table 2). These results comply with the previous finding that breathing exercises were most frequently used among different coping strategies for MPA by student musicians (Studer et al., 2011). Previous literature suggests that relaxation techniques such as deep breathing can reduce MPA by helping musicians to control physiological MPA symptoms (Su et al., 2010; Stern et al., 2012; Zhukov, 2019). Since the recognition of physiological MPA symptoms such as increased heart rate peaked in P2 (Figure 1C), musicians may have employed the breathing strategy most frequently during this time period to relieve these symptoms.

In addition, six participants reported engaging in low- or moderate-intensity exercise (e.g., stretching, jogging) in P2 (Table 2), as a pre-performance routine on the day of a public performance. For example:

“I take light exercise such as jogging because it can help me perform well.” (a student)

Previous studies show that exercise before a public performance or on a daily basis can reduce MPA by attenuating stress responses such as increased heart rate and muscle stiffness (Taylor and Wasley, 2004; Rocha et al., 2014). Participants of the present questionnaire survey may thus have attempted to relieve these symptoms reported in P2 and P3 by employing the exercise strategy. Other popular physical strategies in P2 included keeping the hands/body warm, wiping sweat, and sleep/rest, all of which would be useful for coping with other physiological MPA symptoms (i.e., cold hands/body, perspiration, and sleep disturbances).

The interview data indicates that musicians employed physical strategies most frequently in P2. Notably, all five students and two out of the three professional pianists reported that they stretched various body parts (e.g., neck, shoulders) before public performance. For example:

“I do stretches a lot. Well, I stretched the neck, shoulders, and trunk… I was moving around a lot.” (a student)

The results comply with those of the questionnaire survey, showing that both student and professional musicians were engaged in low-intensity exercise shortly before public performance to reduce muscle stiffness. As in the questionnaire, four students and two professional pianists tried to keep the hands/body warm by using hand warmers and extra clothes. For example:

“I try to get my hands warm with hand warmers.” (a student)

In addition, the breathing strategy was employed by three participants in P2 and three participants in P3, respectively. For example:

“Recently, before a performance, I take a deep breath… pay attention to breathing. It kind of helps.” (a student)

These comments by participants indicate that musicians not only breathe deeply but also attend to the depth and frequency of breathing. As in the questionnaire, participants also reported employing other physical strategies such as wiping sweat. For example:

“I was wiping sweat on my hands with a towel when I was waiting backstage.” (a student)

Compared to students, professionals seemed to be more aware of the importance of maintaining a good physical condition before public performance. In the interview, only professionals mentioned paying attention to their diet and sleep/rest in P1 and P2. For example:

“In my case, I receive acupuncture three or four days before a performance. I then try to achieve the best physical condition on the performance day.” (a professional pianist)

“If I have something in my stomach, I feel uncomfortable… maybe because of acid reflux? So I have lunch but skip dinner before a performance.” (a professional pianist)

“Well, I do not want to get tired or sleepy in the evening [before a performance], so I definitely have a nap. I try to sleep for 30 minutes or less than one hour before leaving home, which means before arriving at the concert hall. Well, if the concert starts at 8 pm, I will surely become sleepy, so I came to have a nap before a concert in the evening, I assume.” (a professional pianist)

These strategies would be helpful in preventing fatigue and gastrointestinal disturbances from negatively affecting public performance. These results are consistent with previous surveys showing that musicians were engaged in specific behaviors to improve their general physical condition (e.g., eating very little, taking a nap) before public performance (Wolfe, 1990; Roland, 1994).

#### 3.2.3. Performance strategies for MPA

The questionnaire data showed that the number of comments on performance strategies peaked in P1, gradually decreasing toward P4 (Figure 2D). The *post hoc* multiple comparisons for performance strategies demonstrated that the number of comments for P1 was significantly greater than that for P3 (*p* = 0.003), P4 (*p* = 0.002), and P5 (*p* = 0.040), respectively. The results indicate that performance strategies for MPA were mainly employed *during the preparation* for public performance. The most popular performance strategy in P1 was the content of practice, with 15 participants mentioning it (Table 2). This complies with the previous finding that the use of practicing techniques was the most frequently used long-term coping strategy for MPA among young musicians (Fehm and Schmidt, 2006). The content of practice strategy reported in the present study primarily involved specific practicing techniques aiming at improving/maintaining playing skills (nine participants), such as playing at a slower tempo and practicing difficult parts of the piece repeatedly. For example:

“I practice the musical piece at a slower tempo.” (a student)

“I repeatedly practice several specific phrases that are vulnerable to mistakes.” (a student)

Eight participants reported practicing coping methods for any accidents or unusual situations that might arise during public performance (e.g., creating rescue plans, playing the piece in a non-ideal environment). For example:

“I practice starting to perform from the middle of the piece just in case I stop playing during a public performance.” (a student)

“I set the room temperature a bit higher during practice, and check if I can conserve my energy till the end of the piece.” (a student)

In addition, three participants mentioned that they try to improve the efficiency and effectiveness of practice in P1.

The most popular strategy in P2 was also the content of practice, with 11 participants mentioning it (Table 2). The content of practice strategy in this time period involved practicing specific phrases, having a rehearsal, double-checking the musical score, and reviewing what they had learned during practice so far. For example:

“I repeatedly double-check the specific phrases which I feel insecure about.” (a student)

The second most popular performance strategy in P1 was the amount of practice, with 11 participants mentioning it (Table 2). Most of them reported increasing the amount of practice during the preparation for public performance. For example:

“As the performance day approaches, I spend more time in practicing the piece.” (a student)

Three participants mentioned employing the amount of practice strategy also in P2. Interestingly, two of them reported that they reduce their practice hours on the performance day, presumably to avoid fatigue and to maintain an ideal physical condition (Ginsborg, 2004; Klickstein, 2009).

In addition, two participants were engaged in mental rehearsal both in P1 and P2 (Table 2). For example:

“I mentally visualize the keyboard and the stage, and play each note in my mind.” (a student)

The results are in accordance with a recent finding that student musicians utilized mental rehearsal during the preparation for public performance and when backstage (Huang and Song, 2021).

In P3, 10 participants reported employing various performing techniques, such as paying attention to the sounds and expressions (Table 2). For example:

“I pay attention to both the melody and the bass line.” (a student)

“I perform as if I am humming the melody in my mind.” (a student)

In P4 and P5, participants mainly reflected on their public performances (five and nine participants, respectively) based on their memories, the audio recording of the performance, or comments from teachers. For example:

“I listen to the audio recording of my performance to consider what I should improve in the next public performance.” (a student)

Corroborating the results of the questionnaire, interview participants employed performance strategies mainly in P1. All eight participants reported employing the content of practice strategy in P1 and P2. The strategy involved various practicing techniques aiming at improving/maintaining playing skills, such as playing at a slower tempo, practicing difficult parts of the piece repeatedly, practicing the right and left hands separately, and audio/video recording their performances. For example:

“I practice playing slowly… really slowly to make sure of all sounds I play. My teacher told me to listen to the sounds carefully.” (a student)

“…I’m aware of [structure]…for example, a section is repeated three times and then the fourth reaches the top. I intentionally practice slower to pay attention to them. I try to understand the structure of the piece through this kind of deformation.” (a professional pianist)

“I practiced a melody with a different rhythm… and the left hand only. Yes, I practiced the left hand only a lot.” (a student)

These practicing techniques are thought to be effective in gaining musical expertise in general (Klickstein, 2009), and would thus help musicians maintain performance quality even in public performance situations. Of these, playing at a slower tempo, which was mentioned most frequently by participants of the questionnaire survey, may particularly play an important role in memorizing music (Allingham and Wöllner, 2022). During practice of a musical piece, musicians’ movements gradually become automatic as the motor learning proceeds. However, when put under the stress of public performance, musicians tend to regain conscious awareness of the movements as in the early stage of motor learning, and this can cause memory slips during public performance (Ginsborg, 2004). If musicians have simulated the conscious control of movements through slow practice, taking both accuracy and expressions into account, they would be able to cope with the altered state of motor processes. Playing at a slower tempo has also been found to induce positive mental states such as mindfulness, concentration, and self-confidence (Allingham and Wöllner, 2022), all of which can contribute to optimal performance.

Reviewing each musical element, by practicing a part of the piece or the right and left hands separately, is also thought to be useful in maintaining memorization (Klickstein, 2009). Practicing difficult parts of the piece repeatedly has actually been found to be the most frequently used short-term coping strategy, and considered helpful, among young musicians (Fehm and Schmidt, 2006). Furthermore, audio/video recording a performance during practice helps musicians to objectively evaluate their performance quality and improve the efficiency of practice (Klickstein, 2009). All in all, these practicing techniques are considered to contribute to preventing MPA symptoms from impairing performance quality onstage.

In P1, three out of the five students had dress rehearsals by trying on the clothes and/or shoes that they intended to wear on the performance day. Interestingly, in the interview, only student musicians reported the need of dress rehearsal. For example:

“Even when I practice at home before a public performance, I wear shoes for the stage performance every time.” (a student)

In contrast, professional pianists felt that their outfits are not likely to be their concern. For example:

“I did [practice with my stage outfits and shoes on] a long time ago, probably until my college days. I do not do it nowadays. I am not sensitive about this….It absolutely does not matter at all.” (a professional pianist)

“I do not wear stage outfits even during rehearsals.” (a professional pianist)

All eight participants noted that opportunities to perform in public would help them boost their self-confidence in future public performances. For example, one professional pianist talked as follows:

“When I get opportunities to play the same piece of music before an audience a couple of times in a month or so… the more I play in public, the more I feel easy. I think it reduces anxiety.” (a professional pianist)

Previous literature also indicates that many musicians are engaged in physical rehearsals or dress rehearsals, often with small audiences, before public performance (Wolfe, 1990; Roland, 1994; Huang and Song, 2021). This kind of contrived performance situation can actually induce MPA symptoms, and thus provide musicians with opportunities for desensitization and context-dependent learning (Ginsborg, 2004; Klickstein, 2009). Moreover, participants of the present questionnaire survey reported practicing coping with potential accidents or unusual situations of public performance during daily practice (e.g., playing the piece in a non-ideal environment). Together, these strategies may have helped musicians to habituate themselves within specific mental and/or physical states experienced during public performance, and establish strategies to deal with these altered states.

In addition to physical practice using the instrument, participants were also engaged in mental rehearsal/practice in P1 and P2. For example, participants would imagine playing certain phrases of a piece, following each note from memory without actually playing. This kind of mental practice helped musicians to reduce their anxiety about memory slips. The following example dialog illustrates how musicians typically conducted mental rehearsal:

“I try to play all pieces in my mind without actually playing.” (a professional pianist)

As participants of both the questionnaire and the interview note, mental rehearsal involves the imaginary rehearsal of a public music performance without overt physical movements (Ginsborg, 2004; Klickstein, 2009; Huang and Song, 2021). Previous literature suggests that mental rehearsal can have various positive effects on music performance, such as improving learning and memory, achieving greater control over negative emotions, enhancing self-confidence and resilience onstage, and focusing attention on the music itself (Ginsborg, 2004). Therefore, the present participants may have attempted to cope with negative feelings, cognitive problems, and impaired motor control during public performance by using the mental rehearsal strategy.

Regarding the amount of practice, all students and professional pianists reported that more practice hours in P1 helped them feel confident. For example:

“I could feel relieved only by the amount of time for practice.” (a student)

“It is important to practice the piece for longer. If I take time for practice, I feel like that the piece of music sticks with me.” (a professional pianist)

In both the questionnaire and the interview, participants reported that they increase the amount of practice during the preparation for public performance. Previous studies indicate that more practice helps musicians to gain self-confidence and relieves MPA symptoms (Zakaria et al., 2013; Biasutti and Concina, 2014). Therefore, the present participants may also have attempted to boost self-confidence by increasing the amount of practice.

Meanwhile, previous literature suggests that longer practice time can also have adverse effects on music performance by increasing the risk of developing playing-related injuries (Passarotto et al., 2023) or by inducing fatigue before public performance (Klickstein, 2009). In the interview, professional pianists reported carefully adjusting the amount of practice toward the performance day, presumably to avoid these adverse effects. For example:

“On the day before a public performance, I do not practice much. I spend less time practicing. I just practice as usual… I finish what I should do by two days before a public performance. Otherwise, I would get tired on the performance day.” (a professional pianist)

Overall, professional pianists tended to make a more systematic plan regarding the preparation for public performance compared to students. It seemed that professionals could precisely predict potential MPA symptoms and the outcomes of their practice behaviors, based on their considerable experience. This may explain why professionals could appropriately adjust their behaviors toward a public performance. For example, a professional pianist talked as follows:

“We have to practice anyway, so I do kind of plan backward [from a public performance], thinking of how I should practice toward the performance…” (a professional pianist)

In P3, musicians tried to pay attention to the sounds and expressions to concentrate on the performance itself. For example, one professional pianist mentioned it as follows:

“I try to follow the melody line in my mind while performing whatever happens.” (a professional pianist)

During public performance, musicians tend to experience drifts in attention, which are mainly caused by task-irrelevant thoughts, factors in the environment, recall of past experiences, and expectation of future experiences (Connolly and Williamon, 2004; Furuya et al., 2021). The present participants may have attempted to avoid drifts in attention during public performance by intentionally paying attention to specific musical aspects such as the melody and the bass line.

In P4 and P5, musicians tended to reflect on their public performances. For example:

“After a public performance, I listen to the audio recording of the performance to reflect on how it was.” (a student)

The result is in accordance with a recent finding that student musicians reflected on their mistakes made onstage after public performance (Huang and Song, 2021). It has been recommended that musicians reflect on each public performance by using resources such as the audio recording of the performance, carefully considering the causes of success or failure (Klickstein, 2009). This process would enable musicians to set an appropriate goal for their next public performance and devise better coping strategies for MPA symptoms.

## 4. Conclusion

This study examined how the experience of mental, physiological, and behavioral symptoms of MPA changes across different time periods around public performance and how musicians cope with the temporal changes in MPA symptoms by conducting a questionnaire survey and a semi-structured interview.

The results indicate that musicians experienced mental symptoms as soon as they began to prepare for public performance and that the experience of mental symptoms peaked shortly before public performance. The mental symptoms mainly consisted of negative feelings such as anxiety, but also included cognitive problems such as memory slips during public performance. The negative feelings seemed to have been induced primarily by the concern over mistakes and the fear of negative evaluation by others. To cope with negative feelings, musicians employed mental strategies such as positive thinking/self-talk and concentration both before and during public performance. It seemed that these mental strategies helped musicians to gain self-confidence and focused attention to music performance. Musicians also attempted to boost self-confidence by increasing the amount of practice and by utilizing both mental and physical rehearsals. To prevent memory slips during public performance, musicians adopted specialized practicing techniques such as mental practice/rehearsal and playing at a slower tempo. The experience of negative feelings was normally replaced by positive feelings such as relief and achievement when musicians went offstage, but for some musicians, this occurred as soon as they started to perform onstage.

The results further demonstrate that physiological MPA symptoms were experienced shortly before and during public performance. Similarly to mental MPA symptoms, the experience of physiological symptoms peaked shortly before public performance. Musicians experienced a variety of physiological symptoms including increased heart rate, perspiration, cold hands, and gastrointestinal disturbances. To cope with these physiological MPA symptoms, musicians effectively employed a variety of physical strategies shortly before public performance. They particularly used the breathing strategy (e.g., deep breathing) and low- or moderate-intensity exercise (e.g., stretching, jogging) in this time period, which have been found to be effective in attenuating physiological stress responses. Musicians also adopted other physical strategies such as keeping the hands/body warm, wiping sweat, and sleep/rest on the performance day to cope with cold hands/body, perspiration, and sleep disturbances, respectively. Furthermore, musicians utilized both mental and physical rehearsals to habituate themselves to the physiological symptoms experienced during public performance.

Behavioral MPA symptoms, in contrast, were experienced mostly during public performance. The most frequently mentioned symptoms were tremor and muscle stiffness, both of which can have detrimental effects on performance quality. To relieve these muscular symptoms, musicians were engaged in low- or moderate-intensity exercise such as stretching on the day of performance. During public performance, some musicians experienced the actual impairment of performance quality. In an attempt to prevent this, musicians adopted a variety of practicing techniques during the preparation for public performance, such as playing at a slower tempo and practicing difficult parts of the piece repeatedly. Musicians also tried to pay attention to specific musical aspects (e.g., sounds and expressions) to improve concentration during public performance. These practicing/performing techniques together seemed to have helped musicians to maintain performance quality onstage.

The comparison of the interview data between student and professional musicians suggest that students tended to experience the three types of MPA symptoms for a longer duration than did professionals, extending from the beginning of the preparation for a public performance until days or even weeks after the performance. Students also perceived their physiological and behavioral symptoms more negatively than did professionals, which could aggravate their anxiety. To cope with their sustained MPA symptoms, students were more likely to rely on self-talk, dress rehearsal, ritualized behaviors, and support from parents or teachers. In contrast, professionals viewed MPA symptoms as natural and unharmful responses that accompany public performance situations. Professionals could thus form positive and realistic thoughts about public performance more naturally. Overall, professionals could precisely predict potential MPA symptoms and the outcome of their practice behaviors based on considerable experience. Based on these predictions, professionals tended to make a systematic plan regarding the preparation for public performance, appropriately adjusting their practice behaviors and physical conditions toward the performance day. These systematic strategies seemed to be a key to effectively preventing and controlling MPA symptoms. From the standpoint of music education, it would be important for educators to understand these differences between student and professional musicians and complement the limited performance experience of students. More specifically, educators should help students to make a more systematic plan to allow them adequate and appropriate preparation for public performance. Educators should also help students to form more positive and realistic thoughts about MPA symptoms. Incorporating these perspectives in music lessons and lectures would lead to more satisfying performance experiences of student musicians.

It is worth noting some limitations of this study. First, the sample size was relatively small both in the questionnaire survey and the semi-structured interview. In particular, we could not compare the data between male and female musicians due to the gender imbalance of the present sample. Since previous studies suggest that female musicians tend to experience higher MPA compared to male musicians (e.g., Hildebrandt et al., 2012; Cornett and Urhan, 2021), future work should recruit a larger sample of musicians to examine whether there are any gender differences in the coping strategies for MPA. Second, the questionnaire survey involved only undergraduate and postgraduate students majoring in music. Future work should examine whether the findings of the questionnaire survey also apply to professional musicians and novice players. Comparisons of the timeline of coping strategies for MPA between musicians with various skill levels would particularly help elucidate the effectiveness of these strategies. Third, most of the participants of the present study were pianists. Therefore, we could not compare the data of pianists with those of other instrumentalists or singers. Since previous literature suggests that MPA symptoms can vary among different types of musicians (Sokoli et al., 2022), future work should investigate how the characteristics of instruments affect coping strategies for MPA. Finally, the questionnaire survey and the interview could not objectively assess the effectiveness of different coping strategies in public performance situations. Therefore, future empirical studies should examine how each strategy influences musicians’ mental and physical states and performance quality.

In summary, the present findings show that mental, physiological, and behavioral symptoms of MPA exhibit differential timelines. That is, the three types of MPA symptoms were found to be experienced by musicians in different time periods around public performance. The data indicate that musicians utilize different coping strategies according to the temporal changes in MPA symptoms. These findings will contribute to the development of more effective coping strategies and intervention methods for MPA, based on the differential timelines of the three types of symptoms. This would ultimately help young talented musicians to achieve optimal performance onstage, and improve their quality of life.

## Data availability statement

The deidentified questionnaire data supporting the conclusions of this article will be made available by the corresponding author. Further inquiries can be directed to the corresponding author.

## Ethics statement

The studies involving human participants were reviewed and approved by National Institute of Advanced Industrial Science and Technology (AIST) Ethics Committee. The participants provided their written informed consent to participate in this study.

## Author contributions

NI designed and performed the study, transcribed dialogues in the interview, and conducted preliminary analyses of the data. YM designed the study, analyzed the interview data, and wrote the manuscript. MY designed and performed the study, analyzed the questionnaire data, and wrote the manuscript. All authors contributed to the article and approved the submitted version.

## Funding

This study was supported by JSPS KAKENHI (Grants JP18K17915 and JP21K11516), awarded by the Japan Society for the Promotion of Science (JSPS) to MY.

## Conflict of interest

The authors declare that the research was conducted in the absence of any commercial or financial relationships that could be construed as a potential conflict of interest.

## Publisher’s note

All claims expressed in this article are solely those of the authors and do not necessarily represent those of their affiliated organizations, or those of the publisher, the editors and the reviewers. Any product that may be evaluated in this article, or claim that may be made by its manufacturer, is not guaranteed or endorsed by the publisher.

## Abbreviations

MPA: music performance anxiety
P1: time period 1 (from the beginning of the preparation for a public performance until the day before the performance)
P2: time period 2 (from the morning of the performance day until shortly before going onstage)
P3: time period 3 (during the public performance)
P4: time period 4 (from shortly after going offstage until the end of the performance day)
P5: time period 5 (from the next day of the performance until shortly before the next public performance)
